# Effect of Recrystallization Annealing on Corrosion Behavior of Ta-4%W Alloy

**DOI:** 10.3390/ma12010117

**Published:** 2018-12-31

**Authors:** Guoqiang Ma, Qiongyao He, Xuan Luo, Guilin Wu, Qiang Chen

**Affiliations:** 1 College of Materials Science and Engineering, Chongqing University, Chongqing 400044, China; maerkkdp@cqu.edu.cn (G.M.); qyhe@cqu.edu.cn (Q.H.); luo_xuan@cqu.edu.cn (X.L.); 2 Southwest Technology and Engineering Research Institute, Chongqing 400039, China

**Keywords:** tantalum alloy, corrosion, microstructure, CSL boundaries, dislocation boundaries

## Abstract

The effect of recrystallization annealing on corrosion behavior of Ta-4%W alloy was studied. It is found that the deformed sample contains high dense dislocations and dislocation boundaries. During annealing, these dislocations and dislocation boundaries are replaced by recrystallizing grains until the alloy is fully recrystallized. Both the anodic dissolution and the cathodic activity is much more blocked. The corrosion potential gradual shift towards negative values and corrosion current density decrease, while polarization resistance increases after annealing, indicating enhanced corrosion resistance of the alloy. Such an enhancement is caused by the increase of low-Σ coincide site lattice boundaries and decrease of dislocations and dislocation boundaries.

## 1. Introduction

Tantalum (Ta) alloys have attracted great interests in many technological applications, such as electronic industry, high-temperature applications, chemical processing and bone repair implants, for their excellent properties of high density, high melting point, good biocompatibility and high corrosion resistance [1,2,3,4,5]. To protect structural materials, for example stainless steels or nickel based alloys from severe corrosion during processing strong corrosion medium, Ta alloys are usually processed into thin layers on surfaces of these materials to achieve corrosion resistance [1,3]. During such processing, crystallographic defects would generate in these alloys and affect its corrosion resistance during such process [6,7].

The corrosion resistance of Ta alloys in H_3_PO_4_, NaCl and KOH solutions has been extensively examined [8,9,10]. Many studies have also been focused on the preparation of Ta films on structural materials to improve their anti-corrosion against sulfuric acid. Ghorbani et al. [11] reported that Ta film can strongly enhance the corrosion resistance of the 316L stainless steel in Ringer’s solution. Wang et al. [12] suggested that the Ta ion implantation is an effective method to improve the cytocompatibility of pure Fe for biomedical applications and only the Ta ion dosage reaching a critical value can significantly increase corrosion resistance in the solution containing SO_4_^2−^. Moreover, Wei et al. [13] coated Ta alloy on the surface of pure titanium and found that the Ta coating shows excellent corrosion resistance in different concentrations of sulfuric acid. However, these studies ignored corrosion resistance of Ta itself since Ta is much superior to other anti-corrosive alloys in sulfuric acid. Robin and de Souza [14,15] observed the corrosion behavior of Ti–Ta and Nb–Ta alloys in sulfuric acid solutions and found the corrosion rates of all materials increase with Ta contents. Friedrich et al. [16] suggested that Ta can be used as a reactor material for high temperature applications up to 360 °C with strongly oxidizing environments. Piotrowski et al. [17] showed that the shape of polarization curves was strongly influenced by the sulfuric acid concentration when electropolishing Ta in sulfuric acid–methanol electrolytes. More recently, Picone et al. [18] reported some corrosion data for Ta alloys in 0.5 M H_2_SO_4_ at 22.7 °C. Nevertheless, the relationship between microstructures and corrosion behaviors of Ta alloy was less investigated; and there is no known study on the effect of deformation and recrystallization annealing on the corrosion behaviors of Ta. Therefore, the aim of present study is to explore the influence of recrystallizing annealing on corrosion behaviors of deformed Ta alloys in sulphuric acid. To do so, potentiodynamic polarization curves tests, electrochemical impedance spectroscopy (EIS) tests, electron backscatter diffraction (EBSD) and transmission electron microscopy (TEM) characterizations were carried out, and the effect of annealing on corrosion behaviors for the alloy was discussed.

## 2. Materials and Methods

The starting material used for this work was Ta containing 4 wt % tungsten (W), which was obtained by electron beam fusion method followed by hot forging. Then the material (initial 5 mm thickness) was cold rolled to 70% reductions (von Mises strain of 1.39). The rolled sheet is 1.5 mm in thickness finally. Samples with sizes of 12 mm long along the rolling direction (RD) and 10 mm wide along the transverse direction (TD) were cut from the rolled sheet, and then annealed at 1200 °C for 10 min and at 1350 °C for 240 min, respectively. The electrochemical measurements were performed in a multi-functional electrochemical workstation (model CS350) by using a conventional three-electrode cell system, i.e., the sample acts as working electrode, 1 cm^2^ platinum sheet as counter electrode and saturated calomel electrode (SCE) as reference electrode. The electrolytic cell prepared for flat sheet has a fixed 5 mm diameter orifice for exposing the sample surface (0.196 cm^2^ area) and 100 mL capacity. To avoid the interference of crevice corrosion, a thin layer 703 sealant was smeared around the orifice. Prior to EIS tests, there was 1000 s waiting time for getting a steady-state condition and then EIS tests were carried out at stabilized open-circuit potential (OCP) within a frequency range of 100 kHz to 10 mHz and with a 10 mV ac voltage signal amplitude perturbation. Potentiodynamic polarization tests were performed at a scan rate of 1 mV/s with scanning potentials varying from −0.6 V_SCE_ to +1.2 V_SCE_ after the EIS measurements. All corrosion tests were performed on the section containing the RD and the TD. To reduce the effect of surface roughness on corrosion performance, all specimens were ground to 5000 grit abrasive papers and cleaned with deionized water before corrosion tests. During electrochemical corrosion testing, specimens were exposed to a 5 wt % H_2_SO_4_ solution at 33 ± 1 °C. Samples were also immersed in a 50 wt % H_2_SO_4_ containing 1 wt % fluoride ions for 7 days to accelerate the corrosion process.

The microstructures of both deformed and annealed samples were characterized by EBSD and TEM techniques. EBSD samples were polished in a mixture of hydrofluoric acid, sulfuric acid (1:9 by volume fraction) at room temperature, and then characterized by using an Oxford AZtec EBSD system [19] equipped on a JEOL 7800F scanning electron microscope (SEM, Akishima, Japan) operating at 20 kV. TEM foils were prepared by conventional twin-jet technique in a mixture of hydrofluoric acid, sulfuric acid and methyl alcohol (1:5:94 by volume) at 243 K, and then observed in a JOEL JEM 2100 TEM operated at 200 kV. Five TEM foils were prepared for each state sample for good statistics. EBSD and TEM characterizations were conducted on the longitudinal section containing the RD and the ND (normal direction) of samples. Depending on the energy and properties, grain boundaries are classified as Σ 1 boundary (low angle boundaries), low-Σ coincidence site lattice (CSL) boundaries with Σ values ranging from 3 to 29, and general boundaries (high-Σ CSL boundaries and the other boundaries) [20]. CSL boundaries were identified following the Brandon criteria [21]. It should be pointed out that only the boundaries with misorientation higher than 2° are marked out due to the resolution of the EBSD technique. Morphologies of corroded surfaces were observed by the SEM.

## 3. Results and Discussion

**Figure 1:**
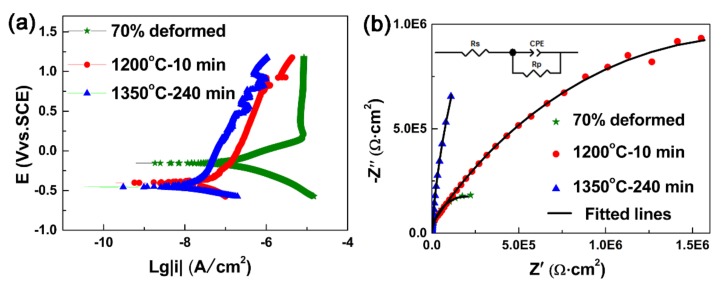
Electrochemical measurements of (**a**) Potentiodynamic polarization curves and (**b**) Nyquist plots.

Figure 1 shows the potentiodynamic polarization curves and Nyquist plots of the Ta alloy. As can be seen in the potentiodynamic polarization curves (Figure 1a), the polarization behavior of deformed sample is much more similar to earlier results [15,18] that is, the anodic and cathodic Tafel behaviors. For the deformed sample, there is a transition point that the anodic slope of plot is changed abruptly and the anodic current increases slightly with increasing more positive the potential. The anodic dissolution continues with a constant rate. However, polarization behaviors of the annealing samples show difference that anodic currents increase gradually with increasing more positive the potential. For both anodic and cathode branches, the current densities of the annealing samples are much smaller than those of the deformed sample one. This indicates that the microstructure is not only affecting anodic dissolution, but also catalysis of the cathodic reaction and the corrosion performance is both governed by the anodic and cathodic reactions. Moreover, there are fluctuations in current for the annealed samples at more positive potential which may be relate to the sudden dissolution events, such as deposited layer drop from the electrode and re-form subsequently. Figure 1b represents the Nyquist plot of different samples in the H_2_SO_4_ solution. For each Nyquist plot, there is only one typical imperfect depressed semicircle. Therefore, there is only one time constant, and there are no intermediate products such as adsorption complex formed on electrode surface. Depending on the shape of Nyquist plot, equivalent circuit model was selected to obtain the polarization resistance (*R*_p_), as shown in the in-set of Figure 1b. *R*_s_ is the resistance of test solution between the reference electrode and working electrode and the constant phase element (CPE), defined as Z = 1/Y_0_ (jw)^-n^ is used to explain non-ideal capacitive response from the interface. It is generally accepted that the diameter of semicircle is related to the *R*_p_ of passive films. Increase of the diameter means increase of corrosion resistance. The corrosion potential (*E*_corr_) and corrosion current density (*i*_corr_) was also obtained by Tafer slope fitting.

The value of *E*_corr_ is −0.16 V for the deformed sample. While the values of *E*_corr_ are −0.40 V and −0.45 V for the sample annealed at 1200 °C for 10 min and the sample annealed at 1350 °C for 240 min, respectively. Therefore, *E*_corr_ of the annealed samples is more negative than that of the deformed one. This indicates that not only the anodic dissolution is much more blocked, but also the cathodic activity is much lower, resulting in lower corrosion rates at *E*_corr_. The *i*_corr_ of the deformed sample is 14 × 10^−8^ A/cm^2^. While the *i*_corr_ is reduced to 7.5 × 10^−8^ A/cm^2^ for the sample annealed at 1200 °C for 10 min, and further decreased to 2.3 × 10^−8 ^ A/cm^2^ after annealing at 1350 °C for 240 min, suggesting that corrosion resistance is enhanced after annealing. The *R*_p_ of the deformed sample is 0.46 × 10^6^ Ω cm^2^. While the values of *R*_p_ are 3.7 × 10^6^ Ω cm^2^ and 7.3 × 10^6^ Ω cm^2^ after annealed at 1200 °C for 10 min and at 1350 °C for 240 min, respectively. It is clear a series of better corrosion resistant behavior, due to the gradual shift of *E*_corr_ towards negative values and gradual decreasing of *I*_corr_ and increasing of *R*_p_.

**Figure 2:**
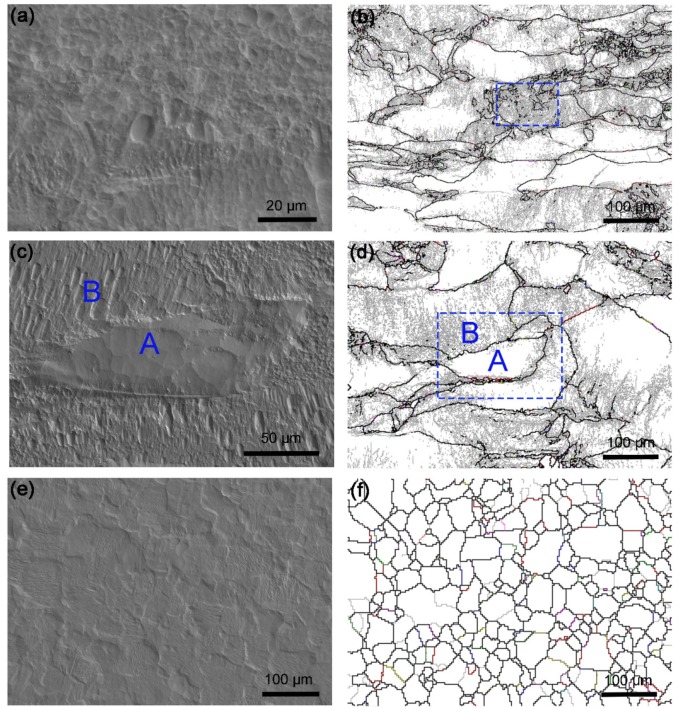
Corrosion SEM images of the (**a**) deformed; (**c**) 1200 °C 10 min annealed and (**e**) 1350 °C 240 min annealed samples, and corresponding grain boundary maps of the (**b**) deformed; (**d**) 1200 °C 10 min annealed and (**f**) 1350 °C 240 min annealed samples, respectively. Less localized corrosion regions are marked by A, and severe localized corrosion regions are marked by B. The dashed boxes indicate the SEM sites.

Figure 2a,c,e shows the SEM images of morphologies for the alloy after accelerated corrosion. The corroded surface was unevenly distributed with localized corrosion for the deformed sample (Figure 2a). While, this phenomenon persists in the sample annealed at 1200 °C for 10 min (Figure 2c). After annealing at 1350 °C for 240 min (Figure 2e), the corrosion surface becomes quite uniform and there are no significant difference between different regions. Figure 2b,d,f show their grain boundary maps. It is seen that severe eroded regions always have deformation induced low angle dislocation boundaries (gray line) or original grain boundaries (black line), while the regions of relatively flat surface are always free of low angle dislocation boundaries, i.e., boundary-free regions. This phenomenon was marked by A and B in Figure 2c,d. Therefore, the corrosion process of the alloy is controlled by microstructural features. During plastic deformation, many dislocations are created and thus dislocation structures are formed. The characteristics of dislocation structures depend on deformation processes and materials [22]. The mechanical energy is stored in form of tangled dislocations and dislocation boundaries. Etch of interfaces immersed in electrolytic solutions was closely related to their mechanical energy. The higher of mechanical energy, the greater corrosion degradation of the region. Since the microstructure of Ta alloy is non-uniform as seen in Figure 2b,d, the mechanical energy changes in different regions. Therefore, corrosion behavior tended to become more localized in the deformed sample and the sample annealed at 1200 °C for 10 min [23]. The inhomogeneity reduced significantly after annealing at 1350 °C for 240 min.

**Figure 3:**
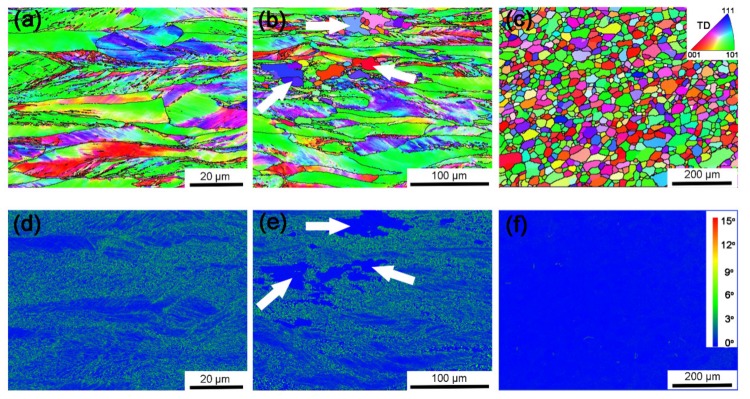
EBSD inverse pole figure (IPF) maps (**a**–**c**) and average misorientation (KAM) maps (**d**–**f**) of (**a**,**d**) 70% deformed; (**b**,**e**) 1200 °C 10 min annealed and (**c**,**f**) 1350 °C 240 min annealed samples, respectively (arrows indicate recrystallizing grains).

**Figure 4:**
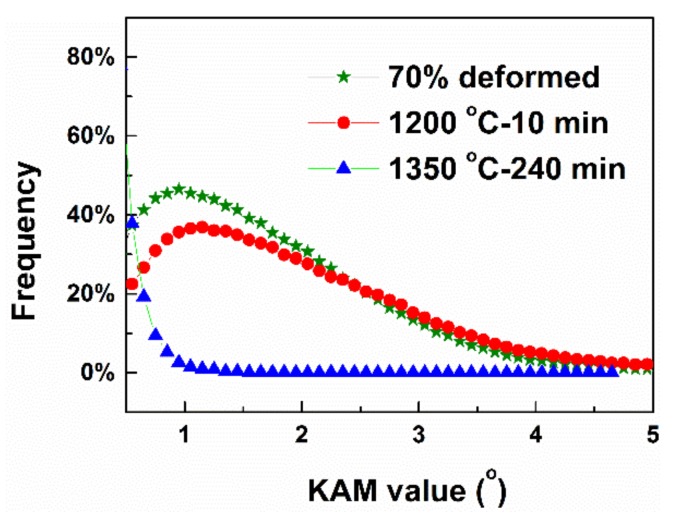
Distribution of KAM value for different samples.

Figure 3 shows the EBSD inverse pole figure (IPF) maps and kernel average misorientation (KAM) map of the alloy. As shown in the IPF map of the deformed sample (Figure 3a), grains are seen to be elongated along RD after rolling. Within these grains, many well-defined dislocation boundaries can be recognized. This is a typical deformation microstructure, which is similar to those in Al, Ni and IF steel [24,25,26,27,28]. These dislocation boundaries have been identified to be geometrically necessary dislocation boundaries (GNBs) [19]. After annealing at 1200 °C for 10 min (Figure 3b), the microstructure is similar to the deformed sample except that a small number of recrystallizing grains are observed (indicated by arrows). These recrystallizing grains have different orientations and distribute in-homogeneously in the deformation matrix, which is related to the heterogeneous deformation structure after rolling. After annealing at 1350 °C for 240 min (Figure 3c), GNBs in the interiors of grains are disappeared and the microstructure is composed of equiaxed recrystallized grains indicating that the sample is fully recrystallized. KAM represents local misorientations between two adjacent points within a grain. It can be applied to characterize dislocation density since the value of KAM directly links to dislocation accumulation extent [29]. As shown in Figure 3d–f, KAM values of majority grains in deformed sample are high. Only a few grains have relatively low KAM, which is coincident with grains boundary-free regions in Figure 2b. However, the KAM values decrease pronouncedly in recrystallized grains (indicated by arrows) in the sample annealed at 1200 °C for 10 min. It should be noted that recrystallization grains prefer to be formed in high KAM regions, shown in Figure 3e. After annealing at 1350 °C for 240 min, all grains show low KAM values. Figure 4 shows the distributions of KAM for individual samples. It is seen that the dislocation density represented by KAM values decreases rapidly after annealing.

**Figure 5:**
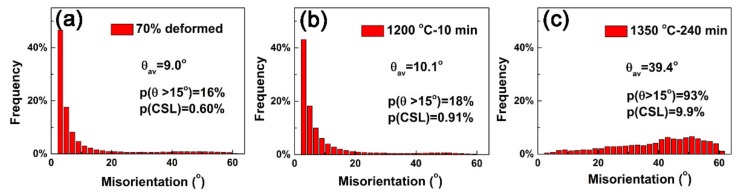
Misorientation distribution of the (**a**) 70% deformed; (**b**) 1200 °C 10 min and (**c**) 1350 °C 240 min annealed samples, respectively.

Figure 5 shows the statistical analysis of boundaries represented by misorientation distributions. The deformed sample as shown in Figure 5a exhibits high contents of Σ1 boundaries, and little change occurs in misorientation distribution after annealing at 1200 °C for 10 min, as shown in Figure 5b. Nevertheless, the sample annealed at 1350 °C for 240 min as shown in Figure 5c shows different misorientation distribution. Most of the low-angle boundaries are disappeared and mainly high-angle boundaries are left. With the increasing annealing temperature and time, both the average misorientation angle and the proportions of high-angle boundaries increase. However, it should be noted that the total number of boundaries reduced after annealing. It is well known that low-Σ CSL boundaries belong to low-energy boundaries comparing to other boundaries and have excellent corrosion resistance [30,31]. Low-Σ CSL boundaries are also parts of high-angle boundaries (misorientation angle ≥ 15°). Thus, higher proportion of high-angle boundaries will result more fraction of low-Σ CSL boundaries [20]. Low-Σ CSL boundaries statistical analysis shows there are approximately 0.6%, 0.91% and 9.9% low-Σ CSL boundaries in the deformed sample and the samples annealed at 1200 °C for 10 min and at 1350 °C for 240 min samples, respectively.

**Figure 6:**
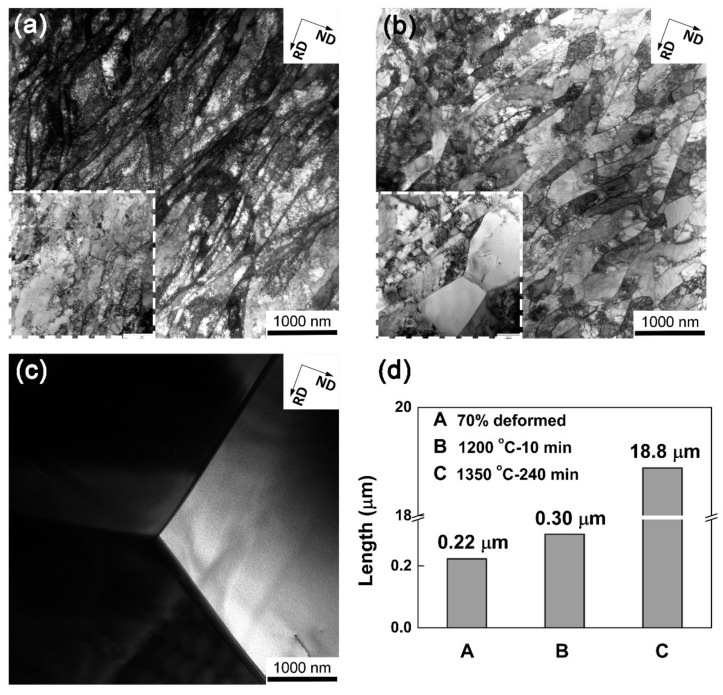
TEM images of the (**a**) deformed sample; (**b**) 1200 °C 10 min and (**c**) 1350 °C 240 min annealed samples; (**d**) distributions of boundary spacing.

Figure 6 presents TEM micrographs of individual samples. In the deformed sample, there are well-defined dislocation boundaries (being operated at edge-on) exhibiting a certain width (Figure 6a). Between these dislocation boundaries, there are large amounts of tangled dislocations. The “boundary-free regions” detected on the grain boundary map of Figure 2b were also be observed by TEM, as shown in the inset of Figure 6a. These regions compose of diffused dislocation boundaries with very small misorientations (typically lower than 2°). After annealing at 1200 °C for 10 min (Figure 6b), the dislocation boundaries however become sharper and better-defined. These changes suggest the occurrence of a recovery process by rearrangement of dislocations in these boundaries. The tangled dislocations that exist in the volumes between these boundaries decreased distinctly. Nevertheless, the most remarkable change observed is the formation of subgrains as shown in the inset of Figure 6b, which can reduce dislocation density significantly. After annealing at 1350 °C for 240 min (Figure 6c), dislocations boundaries disappear and only a few individual dislocations exist. Grain boundaries can be observed clearly since the sample is fully recrystallized. As shown in Figure 6d, after annealing at 1200 °C for 10 min the average dislocation boundary spacing (D_G_) increases slightly [27]. After annealing at 1350 °C for 240 min, the D_G_ increases greatly.

Deformation induced dislocation can be divided into two categories: statistically stored dislocations (SSDs) and geometrically necessary dislocations (GNDs). SSDs are normally in forms of individual dislocations and dislocation tangles caused by random trapping, while GNDs are in forms of dislocation boundaries due to accommodation of lattice rotation [22]. According to Hughes et al. [27,28], large amount of GNDs will be accumulates to form dislocation boundaries (i.e., GNBs) and SSDs will be stored between these GNBs. The dislocation density (ρ_G_) in these GNBs is estimated as:

ρ_G_ = (1.5θ_av_S_V_)/b, (1)

In this equation, θ_av_ is the average misorientation angle and S_V_ is area per volume of boundaries (S_V =_ 1/D_G_ for deformed and 1200 °C-10 min annealed samples, and S_V_ = π/2D_G_ for 1350 °C-240 min annealed sample) [32] and b is Burgers vector (being 0.286 nm for Ta). The calculated ρ_G_ are listed in Table 1. For excellent corrosion resistance, percentages of low-Σ CSL boundaries in high-angle boundaries are also calculated with p(CSL)/p(θ > 15°). The results are shown in the Table 1. Dislocation boundaries can act as fast diffusion paths and corrosion anode comparing to bulk material. The ρ_G_ estimated by equation (1) decreases dramatically after annealing, and the density of SSDs significantly decrease as can be seen from TEM images. It is clear that more high-angle boundaries are converted to low-Σ CSL boundaries in the annealed sample, especially the fully recrystallized sample. High fraction of low- Σ CSL boundaries will keep the boundary diffusivity closer to the bulk diffusivity [33], which can reduce localized corrosion and lead to the formation of a continuous passive film during the corrosion process.

Previous studies have indicated that grain boundaries act as crystallographic defects and thus facilitate corrosion [34,35]. In this paper, SEM observations and EBSD characterizations demonstrate that localized corrosion tend to happen at regions of original grain boundaries and deformation induced dislocation boundaries. The dislocation boundaries observed in EBSD maps correlate to regions with high KAM value and these dislocation boundaries were confirmed in the TEM. However, the regions assumed as “boundary-free regions” by EBSD actually contain diffused boundaries with low misorientations as detected by the TEM. These regions are few with localized corrosion. It means that anodic processes take place in the regions with high dislocation density, while regions with lower dislocation density will be protected by cathodic process during electrochemical corrosion [36]. The low-Σ CSL boundaries also have excellent corrosion resistance due to the coincidence lattices and low boundary energies. After annealing process, internal dislocation density and dislocation boundaries are decreased and the fraction of low-Σ CSL boundaries are increased. 

**Figure 7:**
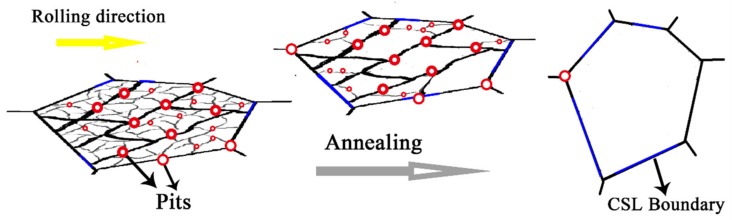
Schematic drawing of the dislocation structure and corrosion behavior of the alloy.

Figure 7 shows the schematic of dislocation structure and corrosion behavior of the alloy. Deformation introduces a large amount of dislocation boundaries (thick line in Figure 7) and tangled dislocations (thin line in Figure 7) between dislocation boundaries. The regions with crystal defects will be high in stored energies and more likely to be accelerated in corrosion. Therefore, pitting is more concentrated in this region [37]. After annealing, the S_V_ of dislocation boundaries and tangled dislocations between dislocation boundaries decrease. The accelerated corrosion is weakened. Moreover, fraction of low-Σ CSL boundaries (blue line in Figure 7) is increased after annealing, which will keep the boundary corrosion behavior closer to the bulk. As a result, corrosion process tends to more uniform. Annealing is a processing method to tailor the structures of Ta, by which the contents of dislocations and dislocation boundaries are reduced and the contents of low-ΣCSL boundaries are increased. Therefore, the increase of corrosion resistance after annealing is related to the increase of low-Σ CSL boundaries and the decrease of dislocation boundaries as well as the tangled dislocations between boundaries.

## 4. Conclusions

The Ta-4%W alloy was cold deformed to 70% reductions in thickness and was annealed. The corrosion resistance of the alloy was evaluated in a 5 wt % sulfuric solution at 33 °C. It has been found that the alloy contains a large amount of dislocation boundaries and tangled dislocations between these boundaries after deformation. After annealing at 1200 °C for 10 min, the density of tangled dislocations decreases greatly, dislocation boundaries become sharper, and recrystallizing grains develop in the matrix. After annealing at 1350 °C for 240 min, the alloy is fully recrystallized and dislocation boundaries in interiors of grains are disappeared. Localized corrosion occurs at regions of original grain boundaries and dislocation boundaries indicating a microstructural feature controlled corrosion process of the alloy. Polarization curves show that corrosion potential gradual shift towards negative values and corrosion current density decrease after annealing. Both the anodic dissolution and the cathodic activity is much more blocked. For every Nyquist plot, there is only one typical semicircle and the polarization resistance increases. It is discussed that the increase of corrosion resistance of the alloy after annealing is caused by the increase of low-Σ CSL boundaries and decrease of dislocations as well as dislocation boundaries.

## Figures and Tables

**Table 1:** The calculate results of ρ_G_ and percentages of low-Σ CSL boundaries.

Sample	ρ_G_ (m^−2^)	p(CSL)/p(θ>15°)
deformed	3.74 × 10^15^	3.75%
1200 °C-10 min	3.08 × 10^15^	5.06%
1350 °C-240 min	3.01 × 10^14^	10.65%
